# Dietary isothiocyanates and anticancer agents: exploring synergism for improved cancer management

**DOI:** 10.3389/fnut.2024.1386083

**Published:** 2024-06-11

**Authors:** Qi Wang, Dan Li, Lihua Liu, Yujuan Shan, Yongping Bao

**Affiliations:** ^1^Norwich Medical School, University of East Anglia, Norwich, United Kingdom; ^2^Department of Nutrition, School of Public Health, Sun Yat-Sen University (Northern Campus), Guangzhou, China; ^3^Department of Nutrition and Food Hygiene, School of Public Health, Wenzhou Medical University, Wenzhou, China

**Keywords:** isothiocyanates, sulforaphane, synergistic interactions, anticancer drugs, chemoprevention, cancer management

## Abstract

Human studies have shown the anticancer effects of dietary isothiocyanates (ITCs), but there are some inconsistencies, and more evidence supports that such anticancer effect is from higher doses of ITCs. The inconsistencies found in epidemiological studies may be due to many factors, including the biphasic dose–response (so called hormetic effect) of ITCs, which was found to be more profound under hypoxia conditions. In this comprehensive review, we aim to shed light on the intriguing synergistic interactions between dietary ITCs, focusing on sulforaphane (SFN) and various anticancer drugs. Our exploration is motivated by the potential of these combinations to enhance cancer management strategies. While the anticancer properties of ITCs have been recognized, our review delves deeper into understanding the mechanisms and emphasizing the significance of the hormetic effect of ITCs, characterized by lower doses stimulating both normal cells and cancer cells, whereas higher doses are toxic to cancer cells and inhibit their growth. We have examined a spectrum of studies unraveling the multifaceted interaction and combinational effects of ITCs with anticancer agents. Our analysis reveals the potential of these synergies to augment therapeutic efficacy, mitigate chemoresistance, and minimize toxic effects, thereby opening avenues for therapeutic innovation. The review will provide insights into the underlying mechanisms of action, for example, by spotlighting the pivotal role of Nrf2 and antioxidant enzymes in prevention. Finally, we glimpse ongoing research endeavors and contemplate future directions in this dynamic field. We believe that our work contributes valuable perspectives on nutrition and cancer and holds promise for developing novel and optimized therapeutic strategies.

## 1 Introduction

Cancer continues to pose a significant global public health challenge, with approximately 20 million new cases and nearly 10 million deaths projected worldwide annually (1). In the United States alone, the 2024 Cancer Statistics report by Siegel et al. estimates 2,001,140 new cases and 611,720 deaths this year. This underscores the ongoing need for advancements in prevention, early detection, and treatment strategies. Despite some declines in mortality rates due to these improvements, the urgency for continued research and better implementation of cancer control measures remains critical to reduce the disease’s overall impact (2).

Within the realm of cancer research, the role of diet, particularly the consumption of dietary isothiocyanates (ITCs) found in cruciferous vegetables, is gaining attention for its potential in cancer chemoprevention and therapy (3, 4). Increasing epidemiological evidence supports an inverse correlation between the consumption of ITC-rich vegetables and cancer risk. Notable studies, such as a prospective analysis in Shanghai, have demonstrated that dietary ITCs are associated with a lowered incidence of lung cancer (5). Similarly, an investigation into cruciferous vegetable intake and mortality in middle-aged adults indicated that higher consumption was linked with lower cancer mortality among men (6). Randomized controlled trials have also suggested that dietary ITCs could be a viable strategy to improve cancer prognosis and survival (7–12). The protective effect of dietary ITCs is particularly pronounced at higher doses or when consumed as part of a cruciferous-rich diet, highlighting the dose-dependent nature of these compounds in cancer prevention (13–15).

Isothiocyanates, such as sulforaphane (SFN), allyl isothiocyanate (AITC), benzyl isothiocyanate (BITC), and phenethyl isothiocyanate (PEITC), are metabolized from glucosinolates and renowned for their potent anticancer properties. Their effectiveness in combating cancer stems from their diverse mechanisms of action, including the modulation of carcinogen metabolism, induction of cell cycle arrest, promotion of apoptotic cell death, activation of cellular defense mechanisms, anti-inflammatory and antioxidant effects, epigenetic regulation, and alteration of various cancer-related signaling pathways (16–21).

Beyond prevention, ITCs show promise in synergizing with conventional anticancer agents, potentially amplifying therapeutic efficacy while minimizing the adverse side effects typically associated with chemotherapy. This synergy is evident through heightened cytotoxic effects on cancer cells, collaborative modulation of drug metabolism and efflux, enhanced drug uptake, and the synergistic induction of apoptotic pathways (17).

One significant impact of isothiocyanates (ITCs) on cancer therapy is their potential to combat chemoresistance, a major obstacle in effective cancer treatment. Chemoresistance, where cancer cells develop mechanisms to resist the effects of chemotherapy, often leads to diminished treatment effectiveness (22, 23). ITCs, notably SFN, have been shown to induce chemosensitization in cancer cells. This is achieved by modulating critical signaling pathways, including MAPK and p53, which can reverse resistance and enhance the efficacy of chemotherapy treatments. Specifically, the activation of the MAPK pathway by ITCs can lead to the upregulation of pro-apoptotic signals. In contrast, the stabilization and activation of p53 by ITCs can halt cell growth and induce apoptosis in chemoresistant cancer cells. These mechanisms highlight the potential of ITCs to serve as valuable adjuncts in cancer therapy, particularly in cases where resistance to conventional chemotherapeutic agents poses a significant challenge (24–28).

Despite their potential, the therapeutic application of ITCs is complex and influenced by their biphasic dose–response (hormetic effect). At low doses, ITCs can promote the growth of both normal and cancer cells, whereas high doses are toxic to cancer cells. Consequently, low doses of ITCs may confer adverse effects on cancer patients, while higher doses lead to beneficial outcomes. This dose-dependent behavior necessitates a nuanced comprehension of ITCs in cancer therapy and the importance of individualized treatment approaches based on specific patient profiles and tumor characteristics (29–31). At low concentrations, ITCs may activate mild oxidative stress, leading to the adaptive activation of protective pathways like Nrf2, enhancing cellular defenses against carcinogens. However, at therapeutic concentrations, ITCs exert pronounced cytotoxic effects on cancer cells, demonstrating their potential as effective anticancer agents. This hormetic behavior underscores the importance of carefully considering dosage in the therapeutic application of ITCs, ensuring that their administration is aligned with an optimal therapeutic window that maximizes anticancer effects while minimizing potential adverse effects.

The selection of SFN as a primary focus in this review is due to its established prominence and effectiveness as a bioactive compound with notable anticancer properties. Extensive research identifies SFN as a potent inhibitor of cancer cell proliferation and an activator of apoptosis in various cancer types. Its distinct mechanisms of action, particularly its inhibition of the nuclear factor kappa B (NF-κB), essential for tumor cell growth and survival, make it a compound of particular interest in cancer chemoprevention studies (32). Furthermore, SFN’s ability to target cancer stem cells, thereby addressing a crucial challenge in cancer therapy, underscores its potential as an anticancer stem cell agent (33). The extensive Research Topic of *in vitro* and *in vivo* studies has confirmed SFN’s effectiveness in fighting various cancers. Its ability to modulate critical signaling pathways associated with drug resistance makes it a promising candidate in the search for effective cancer treatment strategies (34). This powerful combination of its chemopreventive, therapeutic, and synergistic potential renders SFN a subject of paramount importance in the intricate landscape of cancer research.

This review is dedicated to unraveling the complex interplay between ITCs and anticancer agents, shedding light on the synergistic potentials that could transform cancer therapy. Our exploration encompasses the interactions between SFN and other phytochemicals, the combinational effects between SFN and other ITCs, and the additive or synergistic mechanisms of action between SFN and anticancer drugs. The potential benefits of such combinations are numerous, including enhanced therapeutic efficacy through additive or synergistic effects, the potential to overcome chemoresistance, the ability to administer clinically tolerable lower doses of individual agents, and the capacity to mitigate the hormetic effects often associated with ITCs.

### 1.1 Dietary isothiocyanates and cancer

Dietary ITCs, derived from glucosinolates found in cruciferous vegetables such as broccoli, Brussels sprouts, cabbage, and kale, transform into their bioactive forms through the action of myrosinase (35, 36). The transformation of these glucosinolate precursors into their active forms, mediated by myrosinase, facilitates various biological effects (14). The anticancer properties of ITCs have been a subject of extensive research, with epidemiological studies suggesting an inverse relationship between the consumption of cruciferous vegetables and the incidence of cancer (31). These findings have been supported by numerous *in vitro* and *in vivo* studies, which have demonstrated that ITCs can modulate several molecular pathways implicated in carcinogenesis.

One of the crucial mechanisms through which ITCs exert their anticancer effects is by modulating the activity of enzymes involved in the metabolism of carcinogens. Specifically, ITCs inhibit phase I enzymes, such as Cytochrome P450 enzymes (CYP1A1, CYP1A2, and CYP1B1), responsible for pro-carcinogens bioactivation. Concurrently, ITCs induce phase II detoxifying enzymes, including Glutathione S-transferases (GSTs), NAD(P)H Quinone Dehydrogenase 1 (NQO1), and UDP-glucuronosyltransferases (UGTs), which facilitate the excretion of carcinogens, thereby reducing their harmful impact (37).

Sulforaphane (SFN) is an extensively researched ITC with potent anticancer activities demonstrated across various cancer models, including those of the colon, lung, breast, and prostate cancer (14, 34, 36, 38–40). SFN’s actions are multifaceted, encompassing the induction of cytoprotective enzymes, modulation of cell proliferation and apoptosis pathways, and regulation of inflammatory responses. SFN’s mechanisms of action are diverse, including induction of cytoprotective enzymes, modulation of signaling pathways involved in cell proliferation and death, and regulation of inflammatory responses, underlining its therapeutic potential (32). Notably, SFN has been reported to affect the epigenetic regulation of gene expression and to activate the nuclear factor erythroid 2–related factor 2 (Nrf2) signaling pathway, which leads to the induction of antioxidant response elements and the enhancement of cellular antioxidant capacity (14). Moreover, SFN has been identified as one of the most potent naturally occurring inducers of phase II detoxification enzymes, which are crucial for the elimination of carcinogens from the body (36). By orchestrating a multifaceted approach against cancer, SFN stands out as a promising candidate for further exploration in cancer chemoprevention and therapy.

### 1.2 Anticancer mechanisms of dietary isothiocyanates

Dietary ITCs have been shown to modulate various cellular and molecular pathways that contribute to their anticancer effects. These mechanisms encompass a broad spectrum of actions, including the inhibition of cell proliferation, induction of cell cycle arrest, promotion of apoptosis, suppression of angiogenesis, alteration of the tumor microenvironment, and modulation of the pharmacokinetics of anticancer drugs (3, 4, 31).

Among the primary mechanisms ITCs exert their anticancer effects is inhibiting cell proliferation. Research indicates that ITCs can decrease the proliferation of various cancer cell lines, thereby restricting tumor growth potential. This antiproliferative effect is often associated with ITCs’ ability to induce cell cycle arrest, particularly at the G2/M phase. By inducing cell cycle arrest, ITCs prevent cancer cells from entering mitosis, thereby impeding tumor growth and progression (41, 42).

Significantly, SFN has been identified to downregulate the PI3K/AKT/mTOR signaling pathway and upregulate the expression of PTEN, a tumor suppressor gene. This modulation results in the suppressed growth of cancer cells, indicating a vital mechanism through which SFN exerts its anticancer effects. Furthermore, SFN enhances the production of Bax, a pro-apoptotic protein, thereby promoting apoptosis in cancer cells. These additional layers of SFN’s action reinforce its potential as a robust anticancer agent by targeting critical cell survival and death regulators.

Apoptosis, or programmed cell death, is another critical mechanism through which ITCs exert their anticancer effects. ITCs have been shown to induce apoptosis in cancer cells by modulating apoptotic signaling pathways (36, 43). This includes the activation of caspases, which are proteases that play a vital role in the execution phase of cell apoptosis (36).

Angiogenesis, the formation of new blood vessels, is essential for tumor growth and metastasis. ITCs have been reported to exhibit anti-angiogenic properties by inhibiting the formation of new blood vessels in tumors (25, 31). This effect may be mediated by suppressing vascular endothelial growth factor (VEGF) and other angiogenic factors, thereby limiting the supply of nutrients and oxygen to the tumor (31, 44, 45).

The tumor microenvironment, comprising various cell types, extracellular matrix components, and signaling molecules, plays a significant role in cancer progression. ITCs have been shown to modulate the tumor microenvironment, thereby affecting cancer cell behavior (31, 40). For instance, ITCs can modify the inflammatory response within the tumor microenvironment, which may contribute to their anticancer effects (39, 46).

Another important aspect of the anticancer activity of ITCs is their influence on the pharmacokinetics of anticancer drugs. ITCs can affect the metabolism and clearance of drugs, potentially enhancing their efficacy or reducing their toxicity (31, 38). This suggests that ITCs could be used as adjuvants to improve the therapeutic outcomes of conventional anticancer treatments.

Furthermore, ITCs are known to activate the Nrf2 pathway, a crucial regulator of cellular antioxidant responses and detoxification of reactive oxygen species (ROS) (43, 47, 48). This activation leads to the upregulation of antioxidant enzymes and cytoprotective proteins, enhancing cellular defense against oxidative stress, a known contributor to carcinogenesis.

Other key molecular targets or links associated with cancer have been found to be regulated by ITCs, including epithelial-mesenchymal transition (EMT) (49, 50), cancer stem cells and stem cell-like properties (27, 28, 33), microtubule/tubulin polymerization (51–53), mitochondrial biogenesis and dynamics and function (21, 54), autophagy (19, 55, 56), glucolipid metabolism (57, 58), telomerase activity (59, 60) as well as gut microbiota (61, 62).

In short, dietary ITCs exhibit a spectrum of anticancer mechanisms, underscoring their potential as chemopreventive and therapeutic agents. Their multifaceted role in cancer management, including the inhibition of cell proliferation, induction of apoptosis, anti-angiogenic effects, modulation of the tumor microenvironment, impact on the pharmacokinetics of anticancer drugs, and activation of the Nrf2 pathway, highlight their significance.

### 1.3 Hormetic effect of dietary isothiocyanates

The hormetic effect, a dose–response phenomenon marked by low-dose stimulation and high-dose inhibition, constitutes a crucial element in the biological impact of ITCs on cancer management [48, 49]. At lower concentrations (1–5 μM), SFN has been observed to stimulate cell growth in normal cells, potentially aiding in processes such as tissue repair and maintenance. This stimulatory effect on normal cells may render SFN less toxic to normal cells than cancer cells, suggesting a selective action that could benefit therapeutic contexts (30, 63). However, the same compound presents a complex interaction with cancer cells. While low doses of SFN may stimulate certain types of cancer cells, potentially enhancing tumor growth and spread, higher doses (≥ 10 μM) have been shown to inhibit cancer cell proliferation and exert pro-apoptotic effects. This indicates that SFN may either prevent or promote tumor cell growth depending on the dose and the nature of the target cells, underscoring the importance of precise dosing and the consideration of the cellular context in the potential therapeutic use of SFN (30).

The mechanisms underlying the hormetic effects of ITCs are complex and not fully understood. They may entail the modulation of autophagy pathways, as observed by the inhibition of autophagy negating the stimulatory effect of SFN on cell migration. Additionally, the interaction of ITCs with other dietary components, such as selenium, influences their hormetic behavior, with co-treatment enhancing the protective effects of low-dose SFN against free-radical-mediated cell death (30).

Moreover, the hormetic effects of ITCs are influenced by their ability to activate the Nrf2 pathway, which plays a pivotal role in cellular defense against oxidative stress and various carcinogens. The activation of Nrf2 by ITCs can lead to cytoprotective outcomes, and its role in tumor metastasis and growth has also been reported, indicating a dual role of Nrf2 in carcinogenesis, which further complicates the hormetic nature of ITCs (31). In addition, intracellular ROS is altered differentially by low to high doses of ITCs, and its levels ranging from mild to excessive and the time course of ROS production (transient vs. persistent) can differently determine cell fates and carcinogenesis, thus possibly constituting another core mechanism of ITCs’ hormesis (30).

**Figure 1:**
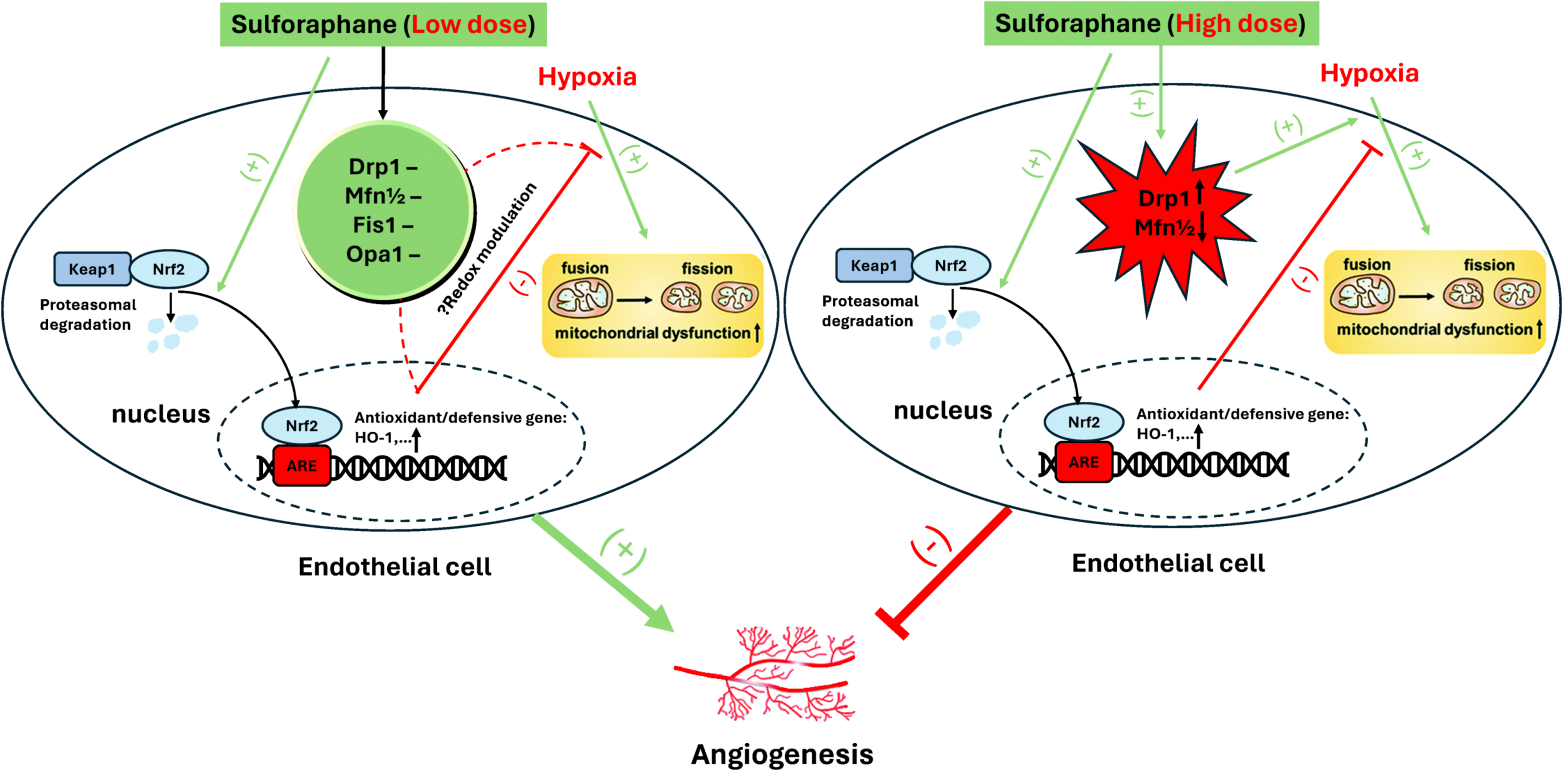
Proposed mechanism of biphasic effect of SFN on angiogenesis in hypoxia. Reproduced from Ref. (64) with permission from the Royal Society of Chemistry.

Interestingly, angio-hormetic effects of ITCs have also been found in our previous studies, using tumor cells and 3-D human umbilical vein endothelial cells (HUVECs) with pericyte co-culture models in normoxia and hypoxia (64). In particular, under hypoxia, a dose-dependent biphasic angio-regulatory effect of SFN was observed and ascribed to mito-hormetic mechanisms that involve an integrated modulation of Nrf2 and alteration of mitochondrial dynamics by SFN. Specifically, Nrf2 activation by low doses (1–5 μM) of SFN can protect against hypoxia-evoked mitochondrial injury and fission, thus boosting the angiogenic capacity of HUVECs. However, mitochondrial fission induced by high doses (≥ 10 μM) of SFN through the regulation of Drp1 and Mfn1/2, coupled with the aggravated mitochondrial injury, may overwhelm the Nrf2- defense dependent beneficial effect, hence mediating anti-angiogenesis. A schematic diagram of the molecular mechanism is shown in Figure 1.

In summary, the hormetic effects of dietary ITCs present a complex interplay between beneficial and potentially adverse outcomes determined by the specific dose and cellular context. While low doses may stimulate normal cellular functions, they may also promote angiogenesis and tumorigenesis in cancer cells. Therefore, combining ITCs with chemotherapeutic agents offers a promising strategy to enhance their anticancer potential while mitigating the risks associated with hormesis. Further research is essential to understand the mechanisms driving the hormetic effects of ITCs fully and optimizing their application in cancer management.

## 2 Combinational use of dietary isothiocyanates and anticancer agents

Combinational therapy marks a significant advancement in cancer treatment and prevention by harnessing the synergistic effects of multiple therapeutic agents to boost efficacy, reduce side effects, and counteract drug resistance (65). The integration of dietary ITCs, especially SFN, with both conventional and non-conventional anticancer agents has paved new pathways for enhancing the effectiveness of cancer management. This section delves into the synergistic potential of SFN when used in conjunction with established chemotherapy drugs and other phytochemicals. Research indicates that SFN can enhance the efficacy of chemotherapeutic agents such as cisplatin and doxorubicin (DOX) (66–68). This enhancement is attributed to its role in modulating drug sensitivity, inducing cell cycle arrest and influencing drug efflux transporters (40, 66). Additionally, SFN’s capacity to intensify drug-induced apoptosis and suppress survival pathways in cancer cells contributes to this synergistic effect. This interaction may permit lower dosages of chemotherapeutic drugs, potentially reducing adverse side effects and slowing the development of drug resistance.

The incorporation of ITCs into cancer treatment regimens holds the potential to not only improve therapeutic outcomes but also to mitigate the toxicological impact of chemotherapy. SFN serves a dual role as both a chemosensitizer and a protective agent against drug-induced toxicity, thus paving the way for cancer treatment strategies that are both more tolerable and effective (68). Nevertheless, determining the optimal dosage and the precise combinations of ITCs with anticancer agents is essential to balance therapeutic advantages against potential risks (31). The assessment of combinational effects is often quantified using the combination index (CI) method, where CI values either less than 1, equal to 1, or greater than 1 indicate synergism, additivity, or antagonism, respectively (17, 66, 69). These values provide a quantitative measure of the interaction between compounds, guiding the optimization of combinational treatments for improved efficacy and reduced toxicity.

Since 2006, over 80 published studies on SFN’s combination with other anticancer agents have been extensively evaluated in many reviews. In this article, we specifically focus on more recent publications within the past six years.

### 2.1 Interactions between sulforaphane and other phytochemicals

The combinatorial use of dietary ITCs, such as SFN, with other phytochemicals has garnered significant interest in the field of cancer management. This interest is predicated on the hypothesis that such combinations may exert synergistic effects, thereby enhancing the efficacy of anticancer strategies. This section evaluates recent studies that have tested combinations of SFN with other phytochemicals for enhanced anticancer efficacy. A summary of all relevant studies is presented in Table 1.

**Table 1:** Summary of studies on combinational use of SFN and other phytochemicals.

Combination agents	SFN Dosage	Other phytochemicals Dosage	Cancer types	Study models	Combination index (CI) (Chou-Talalay method)	References
SFN + withaferin A (WA)	5.0 μM SFN	1.0 μM WA	Breast cancer	MCF-7 and MDA-MB-231 cells	MCF-7 CI = 0.7 MDA-MB-231 cells CI = 1	(70, 71)
SFN + genistein (GEN)	2–15 μM SFN	5–25 μM GEN	Breast cancer	MCF-7 and MDA-MB-231 cells, C3 (1)-SV40 Tag transgenic mouse model	CI range: >0.1 to <0.7	(72)
SFN + GEN + sodium butyrate (NaB)	0–10 μM SFN	0–25 μM GEN and 0–5 mM NaB	Breast cancer	MCF-7 and MDA-MB-231 cells	CI range: >0.06 to <0.47	(73)
SFN + quercetin + lycopene + curcumin	2.5 μM SFN	2 μM lycopene, 25 μM quercetin, and 10 μM curcumin	Colon cancer	colon epithelial (CCD841 CoTr), and colon cancer (HT-29, LS174T) cells	Not listed	(74)
SFN + curcumin+ Dihydrocaffeic Acid (DA)	5–30 μM SFN	5–35 μM curcumin and 5–80 μM DA	Colon cancer	HT-29, Caco-2 and healthy colon cell	HT-29: CI = 0.7 (SFN: DA 1:1) at 90% cytotoxicity; CI > 1 (SFN + curcumin) Caco-2 CI = 0.9 (5uM SFN + 35uM curcumin+5uM DA)	(75)
SFN + luteolin (LUT)	0.12–0.62 μM SFN	2.5–12.5 μM LUT		Raw 264.7 macrophages	CI < 0.87	(76)
SFN + epigallocatechin-3-gallate (EGCG)	0–15 μM SFN. For *in vivo* study, 26% SFN (w/w) in food	0–30 μM EGCG. For *in vivo* study, 0.5% EGCG (w/v) in drinking water	Breast cancer	MCF-7, MDA-MB-231, MDA-MB-157 and MCF-10A cells, transgenic mice	Not listed	(77)

Royston et al. uncovered a compelling synergy between Sulforaphane (SFN) and Withaferin A (WA) in combating breast cancer cells (70, 71). WA, from Indian winter cherry, combined with SFN, significantly inhibited cell viability and induced apoptosis in MCF-7 and MDA-MB-231 cells. This combination notably suppressed histone deacetylase (HDAC) expression and influenced DNA methylation transferase (DNMT) activity, tilting gene expression, and favoring pro-apoptotic pathways. The research concluded that even at low concentrations, the WA and SFN duo could effectively hasten cancer cell demise and modulate critical epigenetic modifiers, showcasing a promising pathway for cancer treatment. The integration of SFN with genistein (GEN), another soy-derived DNMT inhibitor, showed a similar impact. The combination treatment showed enhanced efficacy in inducing apoptosis and reducing colony formation in breast cancer cell lines. SFN exhibited strong synergism with GEN (CI < 0.7), especially at specific concentrations (5 μM SFN and 10 μM/15 μM GEN). This synergistic effect extended to promising *in vivo* results, demonstrating potential for mammary cancer prevention and treatment in transgenic mice (72). Furthermore, a tri-combination involving SFN, GEN, and sodium butyrate (NaB) resulted in even more pronounced synergistic inhibition in breast cancer, reaching a minimum CI value of 0.06 in MCF-7 cells (73).

Two other studies have explored the synergy between SFN and complex phytochemical regimens. The first study by Langner et al. (74) investigated a blend (referred to as MIX) of lycopene, SFN, quercetin, and curcumin. It revealed an additive effect in the reduction of colon cancer cell proliferation. The MIX impaired mitochondrial function, instigating cytotoxicity in cancer cells without harming normal cells. Notably, the MIX also enhanced the antiproliferative effects of chemotherapy drugs such as 5-fluorouracil (5-FU) and cisplatin, suggesting its potential as a chemotherapy adjunct. The study highlighted the chemopreventive action of these compounds, particularly when used in combination, and their selective toxicity toward cancer cells, ensuring safety for normal colon epithelial cells and suggesting their suitability for daily dietary inclusion. The second study investigated the synergistic effects of SFN with curcumin and dihydrocaffeic acid (DA) against colon cancer cells (75). Interestingly, the combination of SFN and curcumin showed a relatively high antagonistic effect (CI between 2.5 and 3). At the same time, the SFN + DA (1:1) combination showed significant cytotoxicity and was more selective toward HT-29 colon cancer cells than healthy cells. The CI value of SFN + DA is 0.7, implying a significant synergistic effect. The study suggested that the mechanisms behind the synergistic effects might involve the modulation of oxidative stress, cell cycle arrest, and apoptosis-related pathways.

Moreover, the anti-inflammatory properties of SFN were amplified when combined with luteolin (LUT). The combined treatments led to a more potent inhibition of nitric oxide (NO) production and a reduction in pro-inflammatory protein expression related to the NF-κB pathway and STAT3 activation (76). This synergistic effect not only suppresses pro-inflammatory cytokines but also diminishes oxidative stress, showcasing significant therapeutic promise for inflammatory-related conditions like cancer.

In addition, research by Li et al. on the transgenerational impact of SFN and epigallocatechin-3-gallate (EGCG) from diet on mammary cancer prevention in mice reveals that these compounds can inhibit breast cancer cell growth through epigenetic modifications. The combined consumption of SFN and EGCG demonstrates synergistic advantages in reducing tumor growth and altering tumor-related protein expression in offspring. This suggests that dietary choices can play an important role in cancer prevention through epigenetic pathways (77).

While the evidence for SFN’s synergistic effects with other phytochemicals is compelling, it is crucial to recognize the limitations of these findings. Most of the studies are preclinical, and translating these findings to clinical settings requires careful consideration of the pharmacokinetics, bioavailability, and potential interactions of these compounds in humans. Additionally, the optimal doses and ratios for combination treatments need to be determined for each specific type of cancer, as the efficacy of these combinations may vary depending on the cancer cell type and the individual’s metabolism.

In conclusion, the preclinical evidence suggests that the combination of SFN with other phytochemicals can lead to synergistic effects in cancer management. However, further research is needed to understand the underlying mechanisms of these interactions and establish the clinical relevance of these findings. The use of CI values in these studies provide a quantitative measure of the interactions, with CI values less than 1 indicating synergism. These values are crucial for determining the potential clinical applications of these combinations and for designing future studies to optimize the use of dietary ITCs in combination with other anticancer agents.

### 2.2 Combinational effect between sulforaphane and other isothiocyanates

The exploration of combinational effects between SFN and other ITCs is a promising area of research, aiming to enhance the efficacy of cancer chemoprevention and therapy. For instance, the combination of SFN with PEITC has been shown to exert synergistic effects in inhibiting inflammation, a process closely linked to carcinogenesis (78). This synergism is likely due to the combined induction of phase II/antioxidant enzymes, including heme-oxygenase1 (HO-1) and NAD(P)H:quinone oxidoreductase 1 (NQO-1), which play a crucial role in the detoxification of carcinogens and protection against oxidative damage (78).

Hutzen et al. (79) explored the effects of SFN and BITC on PANC-1 pancreatic cancer cells and MDA-MB-453 breast cancer cells. Notably, BITC inhibited the phosphorylation of STAT3, a protein implicated in cancer cell growth and survival, whereas SFN’s inhibitory effects appeared to be STAT3-independent. Additionally, BITC prevented IL-6-induced STAT3 activation in MDA-MB-453 cells. Combining BITC and SFN proved more effective than either compound alone in reducing cell viability. This combination exhibited an enhanced reduction in pSTAT3 levels and an amplified increase in apoptosis, evident through PARP cleavage.

Furthermore, the combination of SFN with AITC has demonstrated synergistic effects in non-small cell lung carcinoma cells, manifested by increased production of intracellular ROS and concomitant suppression of cancer cell proliferation (17). Moreover, the synergy between AITC and SFN was also observed in cell migration assays, demonstrating the potential of different ITCs used in combination to produce enhanced protective effects against carcinogenesis.

In summary, these findings suggest a potential synergistic relationship between SFN and other ITCs in inhibiting cancer cell growth, migration, and apoptosis. However, the CI values, which quantify the degree of interaction between two agents, are not explicitly discussed in these studies. Furthermore, these investigations are constrained to *in vitro* contexts; thus, translation to *in vivo* models is a requisite for advancing these preliminary findings. Future research should encompass the deployment of suitable models to evaluate and validate these *in vitro* observations.

### 2.3 Synergisms between sulforaphane and anticancer drugs

The investigation into dietary ITCs, such as SFN, and their combinatorial use with anticancer drugs is at the forefront of oncological research. This focus is driven by the premise that ITCs can amplify the therapeutic effects of standard chemotherapy agents, thereby improving their anticancer properties while potentially diminishing the associated toxicities. This section provides an analysis of recent investigations that have examined the combined use of ITCs with chemotherapeutic agents to enhance anticancer efficacy, focusing specifically on SFN. The assessment considers augmented efficacy, identifies potential limitations, and discusses the significance of Combination Index (CI) values. An overview of these studies is provided in Table 2.

**Table 2:** Summary of studies on combinational use of SFN and anticancer drugs.

Combination agents	SFN dosage	Anticancer drug dosage	Cancer types	Study models	Combination index (CI) (Chou-Talalay method)	References
SFN + gefitinib	0–12 μM SFN	0–2 μM gefitinib	Lung cancer	gefitinib-tolerant PC9 cells	Not listed	(80)
SFN + cisplatin (CIS)	0–10 μM SFN	0–20 μM CIS	Ovarian cancer	A2780 and OVcAR cells,	Not listed	(81)
SFN + CIS	0–1.5 μM SFN	30 μM CIS	Ovarian cancer	A2780 and IGROV1 cells and cisplatin resistance A2780/CP70 and IGROV1-R10 cells	Not listed	(82)
SFN + CIS	0.5 or 1 μM SFN	0.5 or 1 μM CIS	Epidermal squamous cell carcinoma	SCC-13 and HaCaT cells	Not listed	(42)
SFN + CIS	0–20 μM SFN	10 or 25 μM CIS	triple-negative breast cancer (TNBC)	MDA-MB-231 and MDA-MB-468 cells	CI < 0.8 (10 μM SFN + 10 μM CIS)	(83)
SFN + 5-fluorouracil (5-FU)	5.4–42.8 μM SFN	6.4–51.5 μM 5-FU	TNBC	MDA-MB-231 cells	0.5 < CI < 0.7	(84)
SFN + 5-FU	5.9–47.3 μM SFN (Caco-2); 6.2–14.4 μM SFN (HT-29)	1.8–14.4 μM 5-FU (Caco-2); 5.3–12.3 μM 5-FU (HT-29)	Colon cancer and Prostate cancer	Caco-2, HT-29, LNCaP, and PC3 cells	Caco-2 CI = 0.7 HT-29 CI = 0.8 PC-3 CI = 1 LNCaP CI = 1.3	(85)
SFN analog, 2-oxohexyl isothiocyanate (2-oxohexyl ITC) + 5-FU	2.6–20.8 μM ITC (Caco-2); 3.5–8.2 μM ITC (HT-29); 2.8–22 μM ITC (PC-3); 7.6–60.8 μM ITC (LNCaP)	1.8–14.4 μM 5-FU (Caco-2); 5.3–12.3 μM 5-FU (HT-29); 2.9–22.8 μM 5-FU (PC-3); 2.0–15.7 μM 5-FU (LNCaP)	Colon cancer and Prostate cancer	Caco-2, HT-29, LNCaP, and PC3 cells	Caco-2 CI < 1 HT-29 CI < 0.5 PC-3 CI = 1 LNCaP CI > 1.1	(69)
SFN analog 4-isoselenocyanato-1-butyl 4-fluorobenzyl sulfoxide (ISC) + 5-FU	2.0–3.0 μM ISC; For *in vivo* study, ISC at the dose of 50 mg/kg	12.9–51.5 μM 5-FU; For *in vivo* study, 5-FU at 100 mg/kg	TNBC	MDA-MB-231 and 4 T1 cells, 4 T1 tumor-bearing female BALB/c mice	0.5 < CI < 0.8	(86)
SFN + 5-FU, folinic acid and oxaliplatin (FOLFOX)	1.25 or 10 μM SFN	0.4 μM 5-FU, 4 μM folinic acid and 0.2 μM oxaliplatin	Colorectal cancer	CX-1 cells	Not listed	(87)
SFN + 5-FU	0.5–30 μM SFN	0.5–30 μM 5-FU	Colon cancer	HCT-15 cells	Not listed	(88)
SFN + Loratadine (LOR)	5 μM SFN;For *in vivo* study, SFN at the dose of 4 mg/kg	0–1,000 μM LOR; For *in vivo* study, LOR at 0.16 mg/kg	Pancreatic cancer	MIA PaCa-2 and Panc-1 cells	Not listed	(89, 90)
SFN + doxorubicin (DOX)	2.2–12.32 μM SFN (MCF-7); 0.37–8.8 μM SFN (MDA-MB-231)	0.14–0.77 μM DOX (MCF-7); 0.007–0.166 μM DOX (MDA-MB-231)	Breast cancer	MCF-7 and MDA-MB-231 cells	MCF-7 CI = 1 MDA-MB-231 CI < 0.76	(67)
SFN + DOX	40 μM SFN; For *in vivo* study, SFN at the dose of 4 mg/kg	50 nM DOX; For *in vivo* study, DOX at 5 mg/kg	Breast cancer	4 T1 cells and 4 T1 tumor-bearing female BALB/c mice	Not listed	(48)
SFN + DOX	0.031–2 μM SFN; For *in vivo* study, SFN at the dose of 1 mg/kg	0.0156–1 μM DOX; For *in vivo* study, DOX at 1.5 mg/ kg	TNBC	MDA-MB-231, and MCF-10A cells, 4 T1 tumor-bearing female BALB/c mice	MDA-MB-231 CI < 0.56 MCF-10A CI > 1	(91)
SFN + DOX	2.5 μM SFN; For *in vivo* study, SFN at a dose of 4 mg/kg	5 μg/mL DOX; For *in vivo* study, DOX at 5 mg/kg	Breast cancer	MCF-7, MDA-MB-231, 13,762 MAT B III and MCF-10A cells; Female Sprague Dawley rats	Not listed	(92)
SFN metabolites + paclitaxel (PTX)	10 μM SFN metabolites	0–40 nM PTX	Non-small cell lung cancer (NSCLC)	A549 and Taxel-resistant A549 cells	Not listed	(93)
SFN + acetazolamide (AZ)	0–80 μM SFN	0–80 μM AZ	Lung cancer	H727 and H720 cells	Not listed	(94)
SFN + PTX	73 μM SFN	3.08 μM PTX	Prostate cancer	PC3, DU145, and VCaP cells	PC3 CI < 0.41	(95)
SFN + clofarabine (ClF)	10 μM SFN	50 nM or 640 nM CIF	Breast cancer	MCF-7 and MDA-MB-231 cells	MCF-7 CI = 1 MDA-MB-231 CI = 0.9	(96)
SFN + gemcitabine (GEM)	6.8/13.7/27.4 μM SFN (HuCCT-1); 8.5/17.1/34.2 μM SFN (HuH28)	0.14/0.28/0.57 μM GEM (HuCCT-1); 0.17/0.35/0.71 μM GEM (HuH28)	Liver cancer	HuCCT-1 and HuH28 cells; BALB/c mice	HuCCT-1 CI < 0.6 HuH28 CI < 0.7	(97)

One of the critical challenges in cancer therapy is the development of resistance to chemotherapeutic agents (22, 23). Studies have shown that SFN can sensitize cancer cells to various anticancer drugs, thereby potentially overcoming resistance mechanisms. For example, SFN has been shown to curb the growth of gefitinib-resistant lung cancer cells by altering the sonic hedgehog (SHH) signaling pathway and reducing the expression of markers associated with lung cancer stem cells. The synergistic combination of SFN and gefitinib markedly decreases cell proliferation, presenting a viable treatment option for lung cancer (80). Similarly, studies by Kan et al. (81) revealed that pairing SFN with cisplatin (CIS) significantly curtails tumor growth and progression in ovarian cancer xenograft models, demonstrating dose-dependent inhibition of cell proliferation and synergistic effects in colony formation and cell cycle assays. Additionally, research by Liu et al. (82) found that SFN reverses cisplatin resistance in ovarian carcinoma cells through DNA damage induction and enhanced cisplatin retention, an effect partially mediated by the upregulation of miR-30a-3p. This microRNA typically downregulated in cisplatin-resistant cells, plays a crucial role in modulating cisplatin sensitivity, underscoring the therapeutic potential of SFN in overcoming drug resistance.

In epidermal squamous cell carcinoma, the SFN-cisplatin combination proved more effective than either agent alone in suppressing cell proliferation, invasion, and tumor formation. This combination was particularly effective against cancer stem cells, often resistant to therapy, suggesting a novel approach to targeting these resilient cell populations (42). The efficacy of dietary ITCs, such as SFN, extends beyond ovarian and epidermal cancers. In triple-negative breast cancer (TNBC), the combination of SFN and cisplatin exhibited synergistic antiproliferative effects. CI analysis using MTT cell proliferation assay data revealed that most data points fell into synergism (CI < 0.8), indicating a favorable interaction between the two compounds. However, certain concentration combinations exhibited an antagonistic effect (CI > 1), signaling potential antagonism at specific concentrations. This highlights the importance of optimizing dosage to achieve the desired synergistic outcome (83).

The synergistic capabilities of SFN extend beyond its combination with cisplatin. When used in conjunction with 5-fluorouracil (5-FU), an increase in effectiveness has been observed in the treatment of triple-negative breast cancer (84). This combination promotes autophagic cell death and premature senescence, potentially mediated by the Nrf2-KEAP1-ARE signaling pathway. Further analysis using the Chou-Talalay method to evaluate SFN and 5-FU across various cancer cell lines, including the colon and prostate, reveals their complex interaction dynamics. In colon cancer cells, synergism was predominantly observed at higher effect levels, whereas in prostate cancer cells, additive effects were prevalent. The LNCaP prostate cancer cell line, however, showed antagonism at all levels, underscoring the variability in drug combination responses among different cancer types (85).

Additionally, a synergistic effect was observed in colon cancer models with the combination of 2-oxohexyl isothiocyanate (a sulforaphane analog) and 5-FU, particularly enhancing cytotoxic activity and leading to apoptosis in HT-29 cells (69). The combination index (CI) values varied across cell lines, suggesting that the nature of interaction depends on the specific characteristics of each cancer cell line. *In vivo* studies further corroborated these *in vitro* results, with a combination of 5-FU and an SFN analog, 4-isoselenocyanato-1-butyl 4-fluorobenzyl sulfoxide (ISC), showing reduced tumor volume and metastasis in a mammary gland carcinoma animal model, indicating potentiated anticancer activity (86). Moreover, SFN’s combination with FOLFOX (5-FU, oxaliplatin, and folinic acid) in highly metastatic human colon carcinoma cells led to a marked decrease in cell viability, enhanced apoptosis, and inhibited spheroid formation while modulating aldehyde dehydrogenase 1 (ALDH1) activity. Interestingly, while SFN alone increased the expression of multidrug resistance protein 2 (MRP2), its combination with FOLFOX normalized ALDH1 activity, suggesting it could counteract FOLFOX-induced resistance mechanisms (87).

Advancements in targeted delivery systems, particularly the co-encapsulation of ITCs and anticancer agents in nanoparticles, have shown promise for enhancing anticancer effects. For instance, nanotechnology-based approaches utilizing lipid-polymer hybrid nanoparticles (LPHNPs) for delivering SFN and chemotherapeutic agents have been explored to improve bioavailability and therapeutic outcomes, highlighting the potential of nanocarriers in optimizing the synergistic effects of SFN and anticancer drugs. Research on EGF-functionalized LPHNPs encapsulating 5-FU and SFN shows potential for improved cancer treatment delivery and outcomes (88). Similarly, the synergy observed between SFN and Loratadine (LOR) in pancreatic cancer chemoprevention, both *in vitro* and *in vivo*, underscores the effectiveness of novel nano formulations (89, 90). Furthermore, the combining SFN with DOX in liposomal nanoparticles enhances cytotoxicity in breast cancer cells, particularly in hormone-resistant types, indicating the nuanced and cancer-specific nature of these synergistic interactions (67).

Another study focused on the immunomodulatory effects of SFN in the context of breast cancer. The co-administration of SFN and DOX was found to attenuate breast cancer growth by preventing the accumulation of myeloid-derived suppressor cells (MDSCs), which are known to inhibit anti-tumor immunity. This combination therapy led to a significant decrease in tumor volume and expansion of MDSCs, alongside an increase in cytotoxic CD8^+^T cells (48). These findings suggest that SFN can reverse the immunosuppressive microenvironment and enhance the efficacy of DOX. Further research using a TNBC animal model has corroborated the anticancer effects and safety of a liposomal formulation containing DOX and SFN. The combination not only inhibited tumor growth but also exhibited cardioprotective, nephroprotective, and hepatoprotective effects, highlighting the potential for reduced systemic toxicity (91). While SFN has been shown to potentially boost the immune system, some studies indicate it might also be associated with a reduction in T cells. This dual effect suggests a nuanced role for SFN in modulating the immune response within the tumor microenvironment, emphasizing the importance of understanding its comprehensive effects on immune cell populations.

The combination of SFN metabolites and paclitaxel (PTX) shows a synergistic effect in inducing apoptosis in A549/Taxol-resistant lung cancer cells (93). SFN metabolites have been shown to disrupt microtubule dynamics, mechanism of action shared with PTX. This disruption enhances PTX-induced apoptosis in the lung cancer cells, suggesting a potential for reduced drug resistance and lower therapeutic doses. Similar results have been shown by the combination of SFN and acetazolamide (AZ) (94). In another study using prostate cancer cell lines, the co-administration of SFN with PTX resulted in a synergistic effect, reducing the proliferation of both androgen-dependent and independent cancer cells and inducing apoptosis. This synergy was quantified by a CI value of less than 1, indicating a potentiated therapeutic effect when both agents were used together (95).

The synergistic interaction between SFN and anticancer drugs is not only limited to enhancing efficacy but also extends to reducing the toxicity associated with chemotherapy. For example, the combination of SFN with doxorubicin has been shown to potentiate the anticancer effects of doxorubicin while attenuating its cardiotoxicity in a breast cancer model (92). This protective effect is attributed to SFN’s ability to activate Nrf2, a key regulator of cellular antioxidant responses, thereby mitigating oxidative stress and inflammation induced by doxorubicin (92). Such findings underscore the potential of SFN to improve the therapeutic index of conventional chemotherapy by enhancing efficacy and reducing adverse effects.

Furthermore, the epigenetic modulation by SFN plays a significant role in its synergistic interactions with anticancer drugs. SFN has proved to induce hypomethylation and upregulation of tumor suppressor genes, thereby sensitizing cancer cells to the effects of chemotherapeutic agents (96). This epigenetic modulation, coupled with SFN’s ability to inhibit HDAC activity, presents a novel mechanism through which SFN can enhance the efficacy of anticancer drugs (97).

In conclusion, the preclinical evidence suggests that the combination of SFN with anticancer drugs holds promise for enhancing the efficacy of cancer treatment regimens. The observed synergistic effects, as evidenced by CI values less than 1, indicate the potential for improved therapeutic outcomes. However, it is important to note that the combinational effects of SFN and anticancer drugs are not always synergistic (67, 69, 85, 91). This highlights the complexity of drug interactions and the need for careful consideration of the specific cellular context when evaluating combination therapies. Further research is essential to address current study limitations and establish clinical relevance.

### 2.4 Mechanisms of anticancer synergy

**Figure 2:**
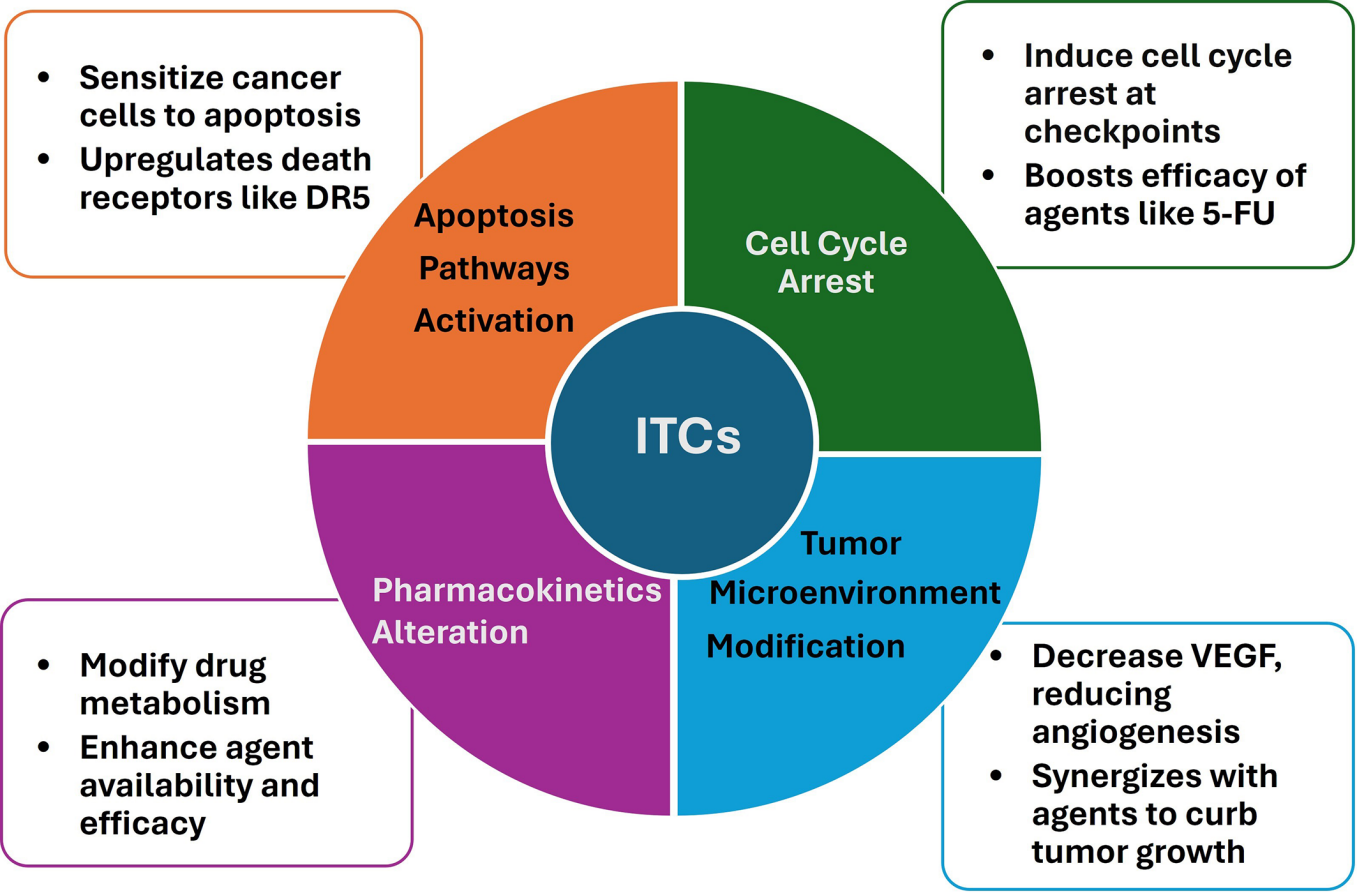
Mechanisms involved in the synergistic interactions between ITCs and anticancer agents.

The combinational use of dietary ITCs with anticancer agents has garnered significant attention due to the potential for enhanced therapeutic efficacy and reduced toxicity. This section delves into the cellular and molecular mechanisms underlying the synergistic interactions between ITCs and various anticancer drugs, which may contribute to improved cancer management (Figure 2).

Apoptosis, a programmed cell death mechanism crucial for removing damaged or unwanted cells, is a common target for ITCs and anticancer drugs. Studies have shown that ITCs, such as SFN and PEITC, can sensitize cancer cells to apoptosis induced by various chemotherapeutic agents (17, 69, 72, 85, 98). This sensitization often involves the upregulation of death receptors (DR), such as DR5, and the activation of both intrinsic and extrinsic apoptotic pathways (32, 66, 81). For instance, the combination of SFN with tumor necrosis factor-related apoptosis-inducing ligand (TRAIL) has been reported to enhance apoptotic signaling in resistant hepatoma cells, suggesting a potential for overcoming drug resistance (81).

The cell cycle is another critical target for the synergistic action of ITCs and anticancer drugs. ITCs have been documented to induce cell cycle arrest at various checkpoints, thereby halting the proliferation of cancer cells (98–100). When ITCs are combined with chemotherapeutic agents that also disrupt the cell cycle, such as 5-FU, the result can be a more pronounced inhibition of cancer cell growth (69, 85). This effect is likely due to the complementary mechanisms of action, whereby ITCs modulate key cell cycle regulators, such as p21, and enhance the cytostatic effects of the drugs (67, 69).

The tumor microenvironment, which includes the surrounding immune cells and extracellular matrix, plays a significant role in cancer progression and response to therapy. One way ITCs influence the tumor microenvironment is by inhibiting angiogenesis, a crucial process for tumor growth and metastasis. This effect is achieved by reducing VEGF secretion (31, 44, 100, 101). This anti-angiogenic effect, coupled with the anti-metastatic properties of ITCs, can synergize with drugs that target the tumor microenvironment, leading to reduced tumor growth and spread (30, 100).

Pharmacokinetics processes, including drug absorption, distribution, metabolism, and excretion in the body, are also affected by the presence of ITCs. These compounds can alter the metabolism of anticancer drugs, potentially increasing their bioavailability and effectiveness (14, 37). For example, ITCs have been shown to modulate the activity of phase II detoxification enzymes, which can influence the bioavailability and effectiveness of anticancer agents (14, 67).

## 3 Risks and considerations in dietary isothiocyanates consumption

While extensive research has focused on the chemopreventive properties of ITCs, there is a growing concern regarding potential risks associated with their consumption, particularly in individuals with undetected tumors. This section aims to underscore these risks and considerations, emphasizing the importance of conducting a comprehensive risk assessment to ensure safety and optimize the benefits of ITC intake.

In this review, we define low doses of SFN as 1–5 μM and high doses as above 10 μM, based on concentrations used in cited studies. It is important to note that these ranges can differ depending on the specific treatment and cellular model. The separation of low and high doses is crucial to avoid overlap and to understand the hormetic effects accurately.

The hormetic effects of isothiocyanates (ITCs) have been a subject of recent studies, indicating that their benefits or risks largely depend on the dose and endpoint of interest (30, 31). Despite their capability to induce apoptosis and hinder cell proliferation in cancer cells, the ability of ITCs to stimulate phase II detoxification enzymes and influence cell cycle regulation could inadvertently promote tumor growth under certain circumstances (37, 98). For instance, while the induction of Nrf2 by ITCs plays a critical role in cellular defense against oxidative stress, its prolonged activation has been associated with tumor promotion and chemoresistance (66, 85). This hormetic nature of ITCs necessitates a careful evaluation of the appropriate dosage for cancer prevention and treatment, as well as the timing of intake relative to other cancer therapies (30).

In combination therapy, the hormetic effect of ITCs may be influenced by the presence of other anticancer agents. Therefore, the defined dosage range of ITCs for their hormetic effect may vary due to the complexity of the mechanisms when ITCs and other anticancer agents work simultaneously. For instance, a lower dose of sulforaphane (SFN) at 2.5 μM can protect cardiac cells from the toxicity of doxorubicin, thereby enhancing the therapeutic window (92). Additionally, up to 1.5 μM SFN can enhance cisplatin sensitivity through the accumulation of intracellular cisplatin in ovarian cancer cells (82). Conversely, 10 μM of SFN can reduce cancer cell resistance to conventional anticancer drugs like cisplatin and paclitaxel, working synergistically to enhance the cytotoxic effects on cancer cells (83, 93). This dual capability underscores the potential of SFN to improve overall therapeutic efficacy. Therefore, a nuanced approach to dose selection in combination therapies is essential to maximize anticancer effects while minimizing potential adverse effects.

Moreover, the influence of ITCs on individuals with undetected tumors remains inadequately understood. There is a potential scenario where ITCs could offer protection against the formation of these tumors. Conversely, they could also inadvertently stimulate the growth of pre-existing yet undiagnosed malignancies (30). This highlights the need for comprehensive risk assessment and personalized dietary recommendations, particularly for individuals at high risk of cancer or those with a family history of the disease.

Another consideration is the variability in individual responses to ITCs, which can be influenced by genetic factors such as polymorphisms in genes related to detoxification enzymes (30). These genetic differences can affect the metabolism and bioavailability of ITCs, leading to variations in their chemopreventive efficacy and potential risks. As such, personalized approaches to ITC intake that take into account genetic predispositions may be necessary to ensure safety and maximize the benefits of these compounds.

In light of these considerations, it is imperative to conduct more research to elucidate the mechanisms underlying the hormetic effects of ITCs and their implications in cancer development and treatment. Studies should aim to determine the optimal doses of ITCs for chemoprevention, identify potential interactions with anticancer drugs, and assess the safety of ITC consumption in individuals with undetected tumors. Additionally, research should explore the genetic factors that influence individual responses to ITCs, with the goal of developing personalized dietary guidelines for cancer prevention.

## 4 Discussion

The comprehensive exploration of ITCs within the context of cancer prevention and therapy showcases a promising avenue in oncology. Particularly, compounds like SFN emerge as potent agents capable of modulating crucial biological processes involved in carcinogenesis, including cell cycle regulation, apoptosis, and anti-angiogenesis. Evidence from epidemiological, *in vitro*, and *in vivo* studies underscores the protective role of ITCs, suggesting their significant contribution to reducing cancer risk and progression. One of the most compelling aspects of ITC research is its potential to synergize with existing chemotherapies. The observed synergistic interactions, as evidenced by CI values less than 1, could potentially amplify the efficacy of chemotherapeutic agents, mitigate adverse side effects, and overcome drug resistance. However, achieving these synergistic effects necessitates careful consideration of dosage and combination strategies to balance therapeutic benefits against potential risks.

In addition, the synergistic effects of ITCs are not limited to interactions with chemotherapeutic agents. Research has indicated that combinations of multiple ITCs or ITCs with other phytochemicals can exert enhanced antiproliferative effects on various cancer cell lines (17, 74). For example, the combined application of lycopene, SFN, quercetin, and curcumin has shown improved antiproliferative potential in colon cancer cells, suggesting that a cocktail of natural compounds may be an effective strategy against tumor growth. These combinations could leverage multiple dietary components to formulate a more practical approach against cancer. Nevertheless, the translation of these preclinical findings into clinical practice requires meticulous attention to factors like bioavailability, pharmacokinetics, and individual metabolic differences.

The potential for personalized treatment strategies based on the synergistic effects of ITCs and anticancer drugs is an exciting prospect. The variability in response to cancer therapies among individuals underscores the need for personalized medicine approaches considering genetic, environmental, and lifestyle factors. The identification of specific biomarkers that predict the response to ITC-based combination therapies could lead to more tailored and effective treatment regimens. Moreover, ongoing clinical trials are investigating the efficacy and safety of ITCs in combination with established anticancer drugs, which will provide valuable data for developing personalized treatment protocols (31).

Despite these encouraging findings, the path to clinical application is fraught with challenges. The hormetic effects of ITCs, characterized by beneficial or adverse outcomes depending on dosage, underscore the need for precise application in therapeutic contexts. The discussion around their potential risks in individuals with undetected tumors, and the variability in individual responses due to genetic factors introduces a layer of complexity. This highlights the necessity for precise dosing, comprehensive risk assessments, and personalized dietary recommendations to maximize the benefits of ITC intake while minimizing potential adverse effects.

Looking forward, the path to fully harnessing ITCs in cancer management is laden with challenges that demand rigorous investigation. To fully leverage the potential of ITCs, prospective studies should prioritize conducting extensive clinical trials to validate the therapeutic efficacy and safety profile of ITCs, particularly in synergy with existing anticancer therapies. A deeper understanding of the molecular mechanisms driving the synergistic effects of ITCs with anticancer agents is essential for the development of more potent and effective combination treatments. Additionally, exploring the potential of ITCs within the realms of targeted therapy and immunotherapy could unveil groundbreaking approaches to cancer treatment. The advancement of nanotechnology-based delivery systems promises to address the current limitations in the bioavailability and stability of ITCs, setting the stage for enhanced cancer prevention and therapeutic strategies. As the field progresses, it is anticipated that these efforts will lead to more effective and less toxic cancer management options, ultimately improving the quality of life and survival rates for cancer patients.

## 5 Conclusion

As we advance our understanding of ITCs in cancer management, the journey toward clinical application is filled with promise and challenges. Future research should focus on validating the therapeutic efficacy and safety of ITCs through clinical trials, particularly in combination with anticancer drugs. Unraveling the molecular mechanisms behind the synergistic effects of ITCs will pave the way for more effective combination therapies. Moreover, integrating ITCs into targeted therapy and immunotherapy could revolutionize cancer treatment approaches.

The advancement of nanotechnology offers promising solutions to the limitations of bioavailability and stability of ITCs, potentially enhancing prevention and therapeutic strategies. As research progresses, these efforts are expected to yield more effective and less toxic options for cancer management, ultimately improving patient outcomes.

The potential of dietary ITCs within a multidisciplinary cancer treatment paradigm holds the promise of a future where cancer therapy is more effective and less burdensome for patients. Harnessing this potential will require a concerted effort across the fields of nutrition, pharmacology, oncology, and biotechnology.

## Author contributions

QW: Conceptualization, Writing – original draft, Writing – review & editing. DL: Conceptualization, Writing – original draft, Writing – review & editing. LL: Writing – review & editing. YS: Writing – review & editing. YB: Writing – review & editing, Conceptualization, Funding acquisition.
